# Uncovering evolutionary and phylogenetic relationships in Glyptothorax species through comparative mitochondrial genomics

**DOI:** 10.1016/j.jgeb.2025.100629

**Published:** 2025-12-09

**Authors:** Somasundaram Iyyappan, Suvadip Ghara, Irfan Ahmad Bhat, Irfan Ahmad Khan, Mohd Ashraf Rather

**Affiliations:** Division of Fish Genetics and Biotechnology, Faculty of Fisheries Ganderbal, Sher-e- Kashmir University of Agricultural Science and Technology, Kashmir 190006, India

**Keywords:** Mitochondrial genome, Comparative genomics, Glyptothorax, Phylogenetic, Evolution

## Abstract

The mitochondrial genome serves as a crucial molecular marker for studying phylogenetic relationships and molecular evolution in fish. Despite their ecological significance in freshwater ecosystems, *Glyptothorax* fishes have limited evolutionary research, with only a few complete mitochondrial genomes reported. The present study examines the complete mitochondrial DNA (mtDNA) of *Glyptothorax cavia*, *G. trilineatus*, *G. annandalei*, *G. sinensis*, and *G. granosus*, with sequence lengths of 16,529 base pairs (bp), 16,539 bp, 16,541 bp, 16,531 bp, and 16,540 bp, respectively. Our comprehensive analysis reveals that most protein-coding genes (PCGs) begin with the ATG codon and terminate with the TAA stop codon, although some exhibit incomplete stop codons (T/TA). The majority of the 13 protein-coding genes (PCGs) display a negative guanine-cytosine (GC) skew, except for the (Skew value: 0.53–0.60) NADH dehydrogenase subunit 6 (ND6) gene. In terms of adenine–thymine (AT) (AT Skew value: 0–0.007), eight PCGs have positive values, while cytochrome *c* oxidase subunit 1 (COX1-), NADH dehydrogenase subunit 3 (ND3), NADH dehydrogenase subunit 4L (ND4L), NADH dehydrogenase subunit 4 (ND4), NADH dehydrogenase subunit 5 (ND5), and cytochrome *b* (Cytb) exhibit negative values. Genetic distance and non-synonymous to synonymous substitution ratio (Ka/Ks) analyses indicate purifying selection acting on the 13 PCGs, with selection pressures potentially influenced by environmental adaptations. Phylogenetic and evolutionary analyses identify *G. sinensis*, *G. annandalei*, and *G. granosus* as closely related species.

## 1 Introduction

Twelve genera are known in the Sisoridae family: *Bagarius, Creteuchiloglanis, Euchiloglanis, Exostoma, Gagata, Glaridoglanis, Glyptosternon, Glyptothorax, Oreoglanis, Pareuchiloglanis, Pseudecheneis, and Pseudexostoma*^1^, ^2^. It is adapted to high-altitude environments and is sporadically dispersed throughout the Tibetan Plateau.^3^ The genus Glyptothorax Blyth, 1860 is among the most varied and widespread sisorid catfishes, notable for its diversity and ecological adaptability.^4^, ^5^ This genus, which is considered monophyletic,^6^, ^7^ as distinguished by its distinctive thoracic adhesive apparatus, which has grooves parallel or oblique to the body's longitudinal axis.^8^ Adapted to fast-flowing waters, members of *Glyptothorax* are rheophilic, typically inhabiting swift hill streams or rapid reaches of larger rivers.^6^, ^9^, ^10^ The Indian subcontinent holds a high diversity of *Glyptothorax* species with 28 valid species reported from the Ganga-Brahmaputra and Barak-Surma-Meghna drainages.^11^, ^12^ Because sisorid fishes share many physical characteristics, classifications based on morphology may be deceptive^13^, ^14^, ^15^. However, previous molecular phylogenetic studies have successfully established well-supported species-level relationships within this family, confirming both the monophyly of Sisoridae and the glyptosternoid group.^13^, ^16^, ^17^, ^18^ However, resolving phylogenetic tree branches often requires information from multiple genes, as data from a single gene is typically insufficient.^19^ Mitochondrial DNA (mtDNA) is a vital genetic resource for understanding and conserving biodiversity and is instrumental in unravelling evolutionary lineages across diverse species.^20^, ^21^ In vertebrates, mtDNA exists as a closed, circular, double-stranded molecule with distinct heavy (H) and light (L) strands, typically spanning 16–20 kb.^22^, ^23^ Mitochondrial DNA is widely used in phylogenetic studies to understand evolutionary relationships among fish species. Its high mutation rate and maternal inheritance make it an effective marker for population genetics, species identification, and biodiversity assessments, evaluations of genetic diversity and population dynamics^24^, ^25^ and phylogeographic investigations.^26^, ^27^ MtDNA consists of 37 genes, including a significant non-coding area that controls transcription and replication, two ribosomal RNAs (rRNAs), 22 transfer RNAs (tRNAs), and 13 protein-coding genes.^28^ The genus *Glyptothorax* holds significant ecological importance in India, primarily inhabiting fast-flowing hill streams and rivers within the Ganga-Brahmaputra drainage system. These catfish contribute to the aquatic ecosystem by supporting the food web and maintaining ecological balance. Their presence is a strong indicator of healthy river systems, making them valuable bioindicators for assessing the ecological health of freshwater environments.^8^ Only limited mitogenomic data and studies on *Glyptothorax* within the Sisoridae family were previously available, and this work provides the first comprehensive comparative analysis of five *Glyptothorax* mitogenome sequences. The findings pave the way for future research on mitogenome structures and molecular marker development, aiding in resolving taxonomic and phylogenetic challenges within *Glyptothorax* and Sisoridae. This baseline data enhances understanding of evolutionary relationships and biogeographic patterns, offering insights into species-specific genetic adaptations crucial for conservation. Furthermore, these results support studies on population genetics, stock identification, and conservation genetics, vital for managing genetic diversity amid threats like habitat degradation and climate change. By highlighting *Glyptothorax*’s role as a bioindicator, this research underscores its ecological importance, advocating for conservation efforts that protect genetic diversity and maintain the health of freshwater ecosystems where these species contribute to ecological balance.

## 2 Materials and methods

### 2.1 Acquisition of sequence Sample

The NCBI (National Center for Biotechnology Information) public database was used to obtain complete mitogenome FASTA samples for five Glyptothorax species: *Glyptothorax cavia* (accession number: NC_034921), *Glyptothorax trilineatus* (accession number: NC_021608), *Glyptothorax annandalei* (NC_045214), *Glyptothorax sinensis* (NC_024672), and *Glyptothorax granosus.*

### 2.2 Sequence annotation and prediction of tRNA secondary structure

The identification of the 37 mitochondrial gene components (Coding genes, tRNA genes and rRNA) for each respective species was carried out using Mitoannotator, a method documented by Zhu et al.^29^ The 2D structure of tRNA were predicted using tRNAscan- SE webserver a method documented by Lowe and Chan.^30^ .

### 2.3 Mitogenome circular genome construction

The CGView Web Server was used to map and graphically show the direction and arrangement of the genes; a tool accessible at https://proksee.ca providing a clear graphical overview of their structure. This platform was utilized to precisely display the gene layout and their respective orientations within the mitochondrial genome.^31^ .

### 2.4 Nucleotide composition and codon usage

MEGA 11 software was used to evaluate the protein-coding genes' (PCGs') base content.^32^ Codon usage bias was evaluated with MEGA 11′s pairwise distance tool.^32^ Skewness in nucleotide composition was calculated using the formulas AT skew (A − T) / (A + T) \) and GC skew (G − C) / (G + C), as described by Perna and Kocher.^33^ .

### 2.5 Phylogenetic analysis

The entire mitogenome sequences were used to build the phylogenetic tree. In MEGA 11, the sequences were aligned with MUSCLE. The out-group employed to root the phylogenetic tree was *Danio rerio*. The tree was constructed using the Neighbor-Joining (NJ) method, and nucleotide substitution distances were estimated using the Kimura 2-Parameter (K2P) model. The robustness of the tree nodes was evaluated using a 1,000-replica bootstrap analysis. After creating the Newick format in MEGA 11, the phylogenetic tree was finally shown using iTOL.^32^ For each divergence dataset, the AliGROOVE was used to get the heterogeneities of sequence divergence.^34^ .

## 3 Result

### 3.1 Mitogenome annotation

**Fig. 1:**
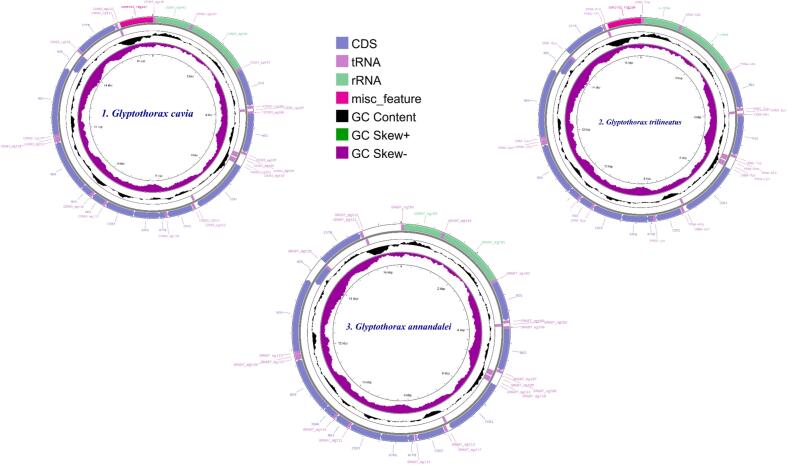

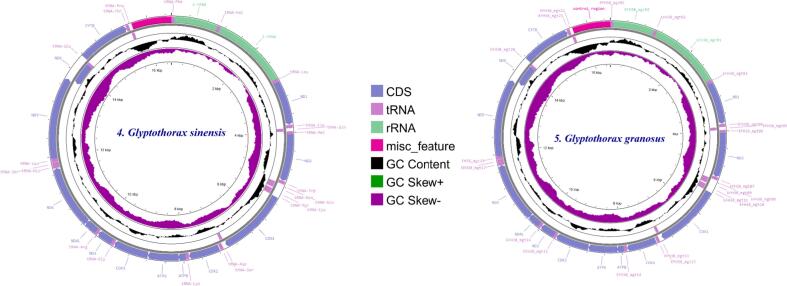
Graphical maps of the mitogenomes gene arrangements of 1) *Glyptothorax cavia,* 2) *G. trilineatus* 3) *G. annandalei* 4) *G.sinensis* 5) *G.granosus.*

The five Glyptothorax species *G. cavia, G. trilineatus, G. annandalei, G. sinensis, and G. granosus* have somewhat different mitochondrial genomes (mt genomes), with relative lengths of 16,529 bp, 16,539 bp, 16,541 bp, 16,531 bp, and 16,540 bp (Table 1). An average of 37 mitochondrial genes, including 13 protein-coding genes (PCGs), 22 transfer RNA (tRNA) genes, and 2 ribosomal RNA (rRNA) genes, are found in each species' mt genome (Fig. 1).

**Table 1:** Annotation details of Respective *Glyptothorax* species.

Species	Annotation											
	Mt Genome Size	tRNAs	rRNAs	PCGs	A	T	G	C	AT Skew	GC Skew	AT	GC
***Glyptothorax cavia***	16,529 bp	22	2	13	31.2	25.9	15.3	27.6	0.092	−0.28	57.1	42.9
***Glyptothorax trilineatus***	16,539 bp	22	2	13	31.3	25.8	15.4	27.5	0.096	−0.28	57.1	42.9
***Glyptothorax annandalei***	16,541 bp	22	2	13	31.3	25.7	15.4	27.7	0.096	−0.28	57	43.1
***Glyptothorax sinensis***	16,531 bp	22	2	13	31.6	26.7	15.4	26.3	0.084	−0.26	58.3	41.7
***Glyptothorax granosus***	16,540 bp	22	2	13	31.1	25.9	15.5	27.5	0.091	−0.27	57	43

### 3.2 Genomic nucleotide profile

Information on the nucleotide makeup of these mitochondrial genomes is given in Table 2. Of the five species of Glyptothorax, *G. sinensis* has the greatest proportion of AT content, followed by *G. trilineatus, G. annandalei, G. granosus, and G. cavia*. *G. cavia*, on the other hand, has the greatest proportion of GC content, followed *by G.annandalei, G.granosus, G. trilineatus, and G. sinensis.* There was an obvious bias against G in three of the species, as evidenced by their low G content. A codon use preference for A and C at the third codon position was also suggested by the noticeably high A and C content at this site. In contrast, the greatest AT content was found in PCGs nucleotide composition by ATPase 8, which was followed by ND5 and ND4. Whereas the GC mean higher value observed in ND4L followed by COX III, ND6 and COXI.

**Table 2:** Combined Coding Sequence (CDS) Nucleotide Composition for five *Glyptothorax* Species.

Species	A%	T%	G%	C%	AT%	GC%
***Glyptothorax cavia***	**28.52**	**27.92**	**15.91**	**27.62**	**56.44**	**43.53**
***Glyptothorax trilineatus***	**28.85**	**28.06**	**15.68**	**27.36**	**56.91**	**43.04**
***Glyptothorax annandalei***	**28.86**	**27.96**	**15.73**	**27.60**	**56.82**	**43.63**
***Glyptothorax sinensis***	**29.32**	**29.16**	**15.71**	**25.82**	**58.48**	**41.53**
***Glyptothorax granosus***	**28.66**	**28.17**	**15.85**	**27.28**	**56.83**	**43.13**

### 3.3 Protein coding Genes, transfer and ribosomal RNA genes

**Fig. 2:**
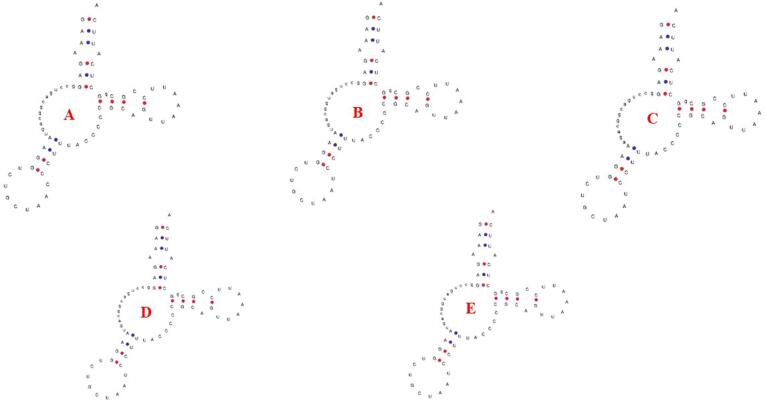
Predicted secondary structures of Serine (Ser) tRNA in five Glyptothorax species: (A) *G. cavia*, (B) *G. trilineatus,* (C) *G. annandalei,* (D) *G. sinensis*, and (E) *G. granosus*. This figure illustrates the conserved and variable regions in the tRNA structures, highlighting species-specific adaptations and structural features in the mitochondrial genomes.

These *Glyptothorax* species have mitochondrial genomes with 29 genes encoded on the heavy (H) strand. These genes include 12 protein-coding genes (PCGs): ND1, ND2, ND3, ND4, ND4L, ND5, COXI, COXII, COXIII, ATP6, ATP8, and Cytb; 2 rRNA genes (12S rRNA and 16S rRNA); and 15 tRNA genes: tRNA-Phe, tRNA-Val, tRNA-Leu, tRNA-Ile, tRNA-Met, tRNA-Trp, tRNA-Asp, tRNA-Lys, tRNA-Gly, tRNA-Arg, tRNA-His, tRNA-Thr, tRNA-Pro, tRNA-Leu, and tRNA-Ser. The light (L) strand contains the remaining genes. Positioned between the tRNA-Pro and tRNA-Phe genes (Fig. 1, Supp Table 1), the regulatory area is bounded at the 5′ end by tRNA-Phe and tRNA-Val and at the 3′ end by tRNA-Leu. Table 1 shows that the sizes of the tRNA genes range from 60 to 87 base pairs. Furthermore, there are minor differences in the lengths of the 12S rRNA and 16S rRNA genes amongst Glyptothorax species. *G. cavia, G. trilineatus, G. annandalei, G. sinensis*, and *G. granosus* all had 12S rRNA lengths of 956, 957, 956, 957, and 959 bp, respectively (Table 3). In contrast, the 16S rRNA, situated between tRNA-Val and tRNA-Leu, measures 1651, 1674, 1636, 1675, and 1675 bp, respectively. This data highlights that the 16S rRNA gene is consistently longer than the 12S rRNA gene. The tRNA ser Secondary structure was analysed due to it is uneven structure. It has lack one arm out of 4 arms. (Fig. 2)

**Table 3:** PCGs genes nucleotide composition of five *Glyptothorax* species.

Species	*Glyptothorax cavia/ G.trilineatus/G. annandalei/ G. sinensis/ G.granosus*			
PCGs	A	T	G	C
**ND1**	28.8/29.5/29.4/30.2/29.3	27.2/27.2/27.2/29.4/26.8	14.1/13.4/13.5/13.5/13.9	29.9/29.8/29.8/26.9/29.9
**ND2**	33.1/33.2/33.3/34.5/32.6	25.5/24.4/24.2/25.1/24.3	11.4/11.6/11.7/12.1/12.4	30/30.8/30.8/28.3/30.6
**COXI**	27.4/27.7/27.7/28/27.5	28.3/28.5/28.1/29.1/28.9	17.7/17.4/17.3/17.2/17.6	26.6/26.4/26.8/25.7/26
**COXII**	30.5/31.3/30.8/31.7/30.5	27.1/28.5/28.7/28.8/28.1	15.8/15.2/15.3/15.2/15.8	26.6/25/25.2/24.3/25.6
**ATPase 8**	37.5/35.1/34.5/37.5/35.7	23.8/24.4/23.8/24.4/24.4	11.9/13.1/13.1/11.9/12.5	26.8/27.4/28.6/26.2/27.4
**ATPase 6**	31.3/31/31/31.5/32.2	26.9/26.5/26.2/28.1/28	12/12.6/12.3/12.2/11.7	29.7/29.9/30.5/27.8/28.1
**COX III**	27.3/27.2/27.2/27.6/27.2	26.1/26.7/26.8/28.1/26.4	16.7/17.3/17.1/17/16.7	29.8/28.8/29/27.4/29.7
**ND3**	26.9/28.9/29.2/29.8/27.8	28.1/28.9/27.2/30.4/29.5	15.2/14.3/14.3/14/15.2	29.8/27.8/29.2/25.8/27.5
**ND4L**	22.9/24.2/23.9/24.6/24.2	25.3/27.9/29.3/29/28.3	15.8/14.8/14.8/15.5/15.2	36/33/32/31/32.3
**ND4**	30/31.2/31.4/30.9/32	27.5/27.5/27.2/28.5/26.8	13.8/13.5/13.5/13.7/13.6	28.7/27.8/28/26.9/28.6
**ND5**	32/31.7/31.9/32/31.3	28/26.8/26.8/27.6/26.7	12.7/12.6/12.5/12.8/12.4	27.3/28.8/28.8/27.6/29.7
**ND6**	14.5/15.6/15.2/14.6/14.6	40.1/39.1/39.7/40.7/39.3	36.4/34.7/35.3/35.1/35.8	9.1/10.6/9.8/9.6/10.2
**CYTb**	28.6/28.5/27.4/27.9/28.8	29.1/28.5/28.4/29.9/28.8	13.4/13.4/13.8/14.1/13.3	28.8/29.7/30.4/28.2/29.1

**Fig. 3:**
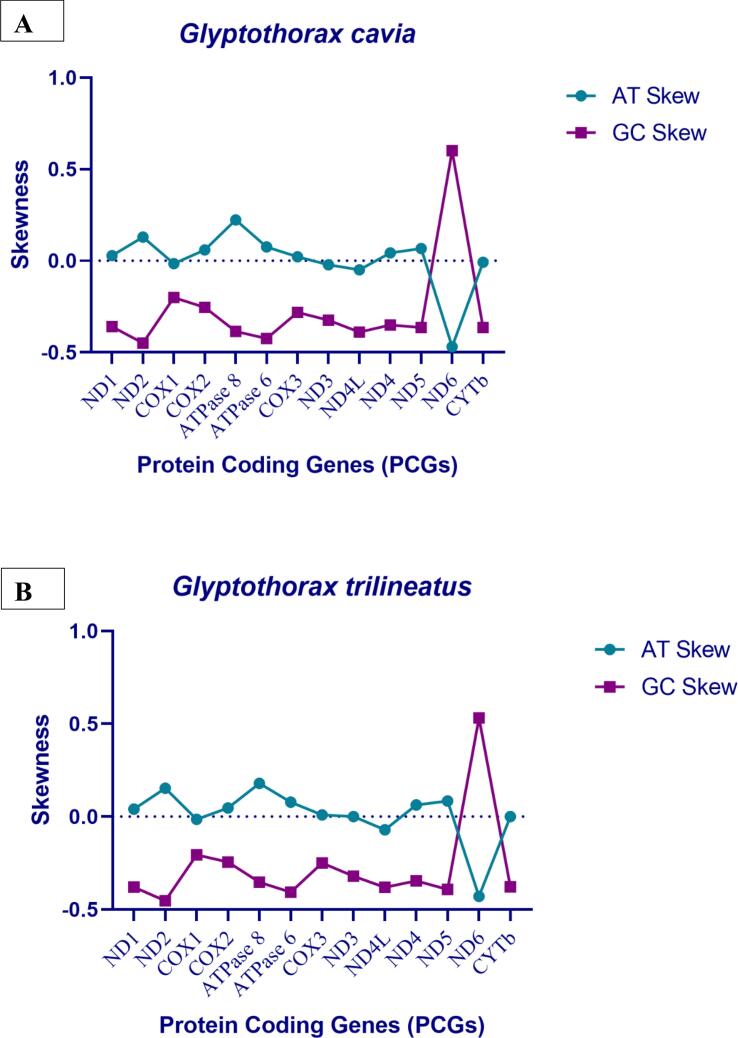

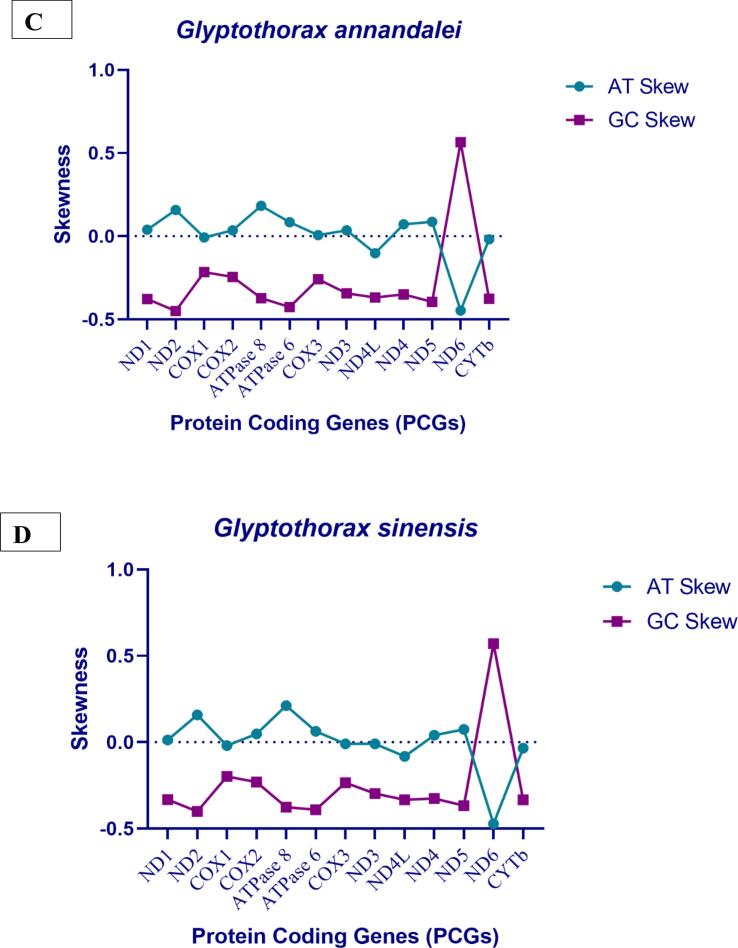

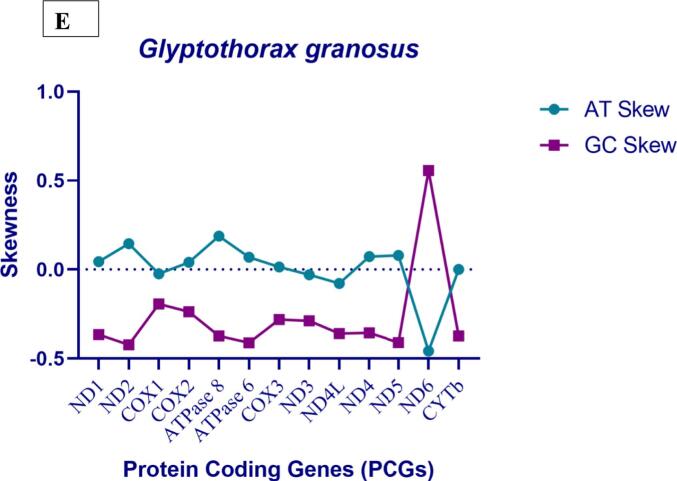
AT and GC skewness analysis of protein-coding genes in Glyptothorax species: (A) *Glyptothorax cavia,* (B) *G. trilineatus*, (C) *G. annandalei,* (D) *G. sinensis*, and (E) *G. granosus.*

### 3.4 Skewness analysis

Nucleotide skewness measurements reveal intriguing patterns across all five Glyptothorax species. Positive AT skews and negative GC skews are consistently observed (Table 1). Among the protein-coding genes, COX1 (AT skew: −0.016), ND3 (−0.0048), ND4L (−0.0762), ND6 (−0.4544), and CytB (−0.0126) show negative AT skews, indicating a thymine bias. The remaining eight protein-coding genes exhibit positive AT skews, with values ranging from 0.0088 (COX3) to 0.1968 (ATP8), suggesting an adenine bias. In terms of GC skew, 12 of the 13 protein-coding genes show negative values, reflecting a cytosine bias. These include genes such as ND2 (−0.4346), ATP6 (−0.4116), ND5 (−0.3852), and ND1 (−0.3614). The only exception is ND6, which displays a positive GC skew of 0.5572, indicating a strong guanine bias (Table 4; Fig. 3).

**Table 4:** Skewness value of Five Glyptothorax species.

Species	*Glyptothorax cavia/ G.trilineatus/G. annandalei/ G. sinensis/ G.granosus*					
PCGs	Mean AT%	GC Mean	AT Skew	GC Skew	Mean AT Skew	Mean GC Skew
**ND1**	57	42.94	0.0285/0.040/0.038/0.013/0.044	−0.359/-0.376/-0.376/-0.331/-0.365	0.0327	−0.3614
**ND2**	58.04	41.94	0.129/0.152/0.158/0.157/0.145	−0.449/-0.452/-0.449/-0.400/-0.423	0.1482	−0.4346
**COXI**	56.32	43.74	−0.016/-0.014/-0.007/-0.019/-0.024	−0.200/-0.205/-0.215/-0.198/-0.192	−0.016	−0.202
**COXII**	59.20	40.80	0.059/0.046/0.035/0.047/0.0409	−0.254/-0.243/-0.244/-0.230/-0.236	0.0456	−0.2414
**ATPase 8**	60.22	39.78	0.223/0.179/0.183/0.211/0.188	−0.385/-0.353/-0.371/-0.375/-0.373	0.1968	−0.3714
**ATPase 6**	58.54	41.36	0.075/0.0782/0.083/0.063/0.0697	−0.424/-0.407/-0.425/-0.390/-0.412	0.0737	−0.4116
**COX III**	54.12	45.36	0.022/0.009/0.007/-0.008/0.014	−0.281/-0.249/-0.258/-0.234/-0.280	0.0088	−0.2604
**ND3**	56.94	42.62	−0.021/0/0.035/-0.009/-0.029	−0.324/-0.320/-0.342/-0.296/-0.288	−0.0048	−0.314
**ND4L**	51.92	47.68	−0.049/-0.0710/-0.101/-0.082/-0.078	−0.389/-0.380/-0.367/-0.333/-0.36	−0.0762	−0.3678
**ND4**	58.60	41.62	0.043/0.0630/0.071/0.040/0.072	−0.350/-0.346/-0.349/-0.325/-0.355	0.0578	−0.345
**ND5**	59	41.04	0.066/0.0837/0.086/0.073/0.079	−0.365/-0.391/-0.394/-0.366/-0.410	0.0777	−0.3852
**ND6**	54.68	45.32	−0.468/-0.429/-0.446/-0.471/-0.458	0.6/0.5/0.56/0.57/0.556	−0.4544	0.5572
**CytB**	57.38	42.84	−0.008/0/-0.017/-0.034/0	−0.364/-0.378/-0.375/-0.333/-0.372	−0.0126	−0.3644

### 3.5 Codon usage

In all five *Glyptothorax* species, the most frequently used codon was AAA (Lys), with the highest usage observed in *G. granosus* (57.6 ‰), followed by *G. annandalei* (57.4 ‰), *G. trilineatus* (55.8 ‰), *G. fokiensis* (54.7 ‰), and *G. sinensis* (54.5 ‰). This was followed by CAA (Gln), with usage values ranging from 51.1 ‰ to 53.1 ‰ across species, and TGA (Trp), with frequencies between 48.3 ‰ and 50.1 ‰. Among amino acids, lysine (AAA) showed the highest codon frequency overall. For leucine, the codon CTA showed notably high usage in *G. granosus* (53.3 ‰), *G. annandalei* (52.7 ‰), and *G. sinensis* (51.5 ‰), while *G. trilineatus* displayed a slightly lower CTA frequency (49.4 ‰). In contrast, the least frequently used codons across all species included TCA (Ser), with values ranging from 3.7 ‰ to 4.5 ‰, GCG (Ala) with 4.0 ‰ to 5.2 ‰, and ACG (Thr), which ranged from 4.1 ‰ to 5.4 ‰, indicating a notable codon usage bias. (Table 5.).

**Table 5:** Codon Usage bias.

Species	*Glyptothorax cavia/ G.trilineatus/ G. annandalei/ G. sinensis/ G.granosus*				
Amino acid	Symbol	Codon	Number	Codon Usage Percentage (%)	Mean Codon Usage Percentage%
**Ala**	A	GCG	11/16/14/14/11	4/8/5/6/4	5.4 %
		GCA	80/56/90/70/100	31/27/33/28/33	30.4
		GCT	64/58/66/71/76	24/28/24/28/25	25.8
		GCC	107/74/106/98/115	41/36/38/39/38	38.4
**Cyst**	C	TGT	40/51/47/38/38	37/41/42/37/42	39.8
		TGC	49/73/65/66/52	63/59/58/63/58	60.2
**Asp**	D	GAT	44/55/43/53/66	33/47/47/54/47	45.6
		GAC	53/62/49/46/74	67/53/53/46/53	54.4
**Glu**	E	GAG	33/54/38/56/56	33/45/34/41/39	42.4 %
		GAA	68/66/73/82/88	67/55/66/59/61	63.2 %
**Phe**	F	TTT	96/81/85/104/96	49/47/47/55/44	48.4
		TTC	101/91/96/84/124	51/53/53/45/56	51.6
**Gly**	G	GGG	42/37/40/46/45	19/24/20/28/21	22.4
		GGA	62/43/53/45/80	28/28/27/27/37	29.4
		GGT	50/31/47/34/40	23/20/24/21/18	23.2
		GGC	68/43/57/40/54	21/28/29/24/25	25.8
**His**	H	CAT	123/117/119/105/103	52/47/52/50/50	49.4
		CAC	122/131/111/105/104	48/53/48/50/50	50.6
**Ile**	I	ATT	159/128/146/149/170	57/59/55/57	57
		ATC	120/89/119/111/113	43/41/45/43	43
**Lys**	K	AAG	67/90/76/87/	28/34/32/33	31.6
		AAA	174/176/165/176/	72/66/68/67	68.4
**Leu**	L	TTG	35/70/37/68/39	6/12/6/10/6	8
		TTA	109/135/129/159/147	19/22/21/24/23	21.8
		CTG	62/81/66/71/69	11/13/11/11/11	11.4
		CTA	158/116/181/169/196	27/19/29/25/30	24
		CTT	122/119/119/111/101	21/20/19/17/16	18.6
		CTC	101/82/91/87/91	17/14/15/13/14	15
**Met**	M	ATG	72/79/66/76/68	35/45/33/37/36	37.2
		ATA	136/95/133/132/123	65/55/67/63/64	62.8
**Asn**	N	AAT	153/167/148/123/125	53/52/49/45/45	48.8
		AAC	138/156/151/148/152	47/48/51/55/55	51.2
**Pro**	P	CCG	48/48/53/52/49	10/9/11/11/10	10.2
		CCA	110/141/108/128/133	23/27/23/28/28	25.8
		CCT	163/173/157/137/130	35/33/33/29/28	31.6
		CCC	148/167/156/148/159	32/32/33/32/34	32.2
**Gln**	Q	CAG	54/86/58/84/56	28/39/27/39/29	32.4
		CAA	140/132/153/130/140	72/61/73/61/71	67.6
**Arg**	R	CGG	26/31/35/28/23	17/25/22/22/18	20.8
		CGA	50/36/42/40/46	32/29/27/32/36	30.4
		CGT	26/22/25/23/19	17/17/16/18/15	17.8
		CGC	52/37/54/35/40	34/19/35/28/31	30.8
**Ser**	S	AGT	65/64/82/51/46	14/12/17/11/10	12.8
		AGC	112/116/106/102/94	24/22/22/22/21	22.2
		TCG	34/38/36/43/32	7/7/7/9/7	7.4
		TCA	89/109/85/93/100	19/21/18/20/22	20
		TCT	78/103/85/98/82	17/19/18/21/18	18.6
		TCC	87/100/89/81/100	19/19/18/17/22	19
**Thr**	R	ACG	33/48/34/38/29	7/11/7/9/7	8.2
		ACA	157/138/154/147/143	33/32/32/35/33	33
		ACT	165/128/151/113/135	34/29/32/27/31	30.6
		ACC	125/124/135/119/131	26/28/28/29/30	28.6
**Val**	V	GTG	27/27/20/29/35	16/18/12/18/17	16.2
		GTA	68/65/64/67/73	40/42/39/42/36	39.8
		GTT	41/34/48/35/45	24/22/29/22/22	23.8
		GTC	32/28/33/29/50	19/18/20/18/25	20.4
**Trp**	W	TGGTGA	45/53/35/48/36100/54/111/82/68	31/50/24/37/3569/50/76/63/65	35.464.6
**Tyr**	Y	TAT	128/125/129/148/114	52/48/54/49/50	50.6
		TAC	120/133/109/153/116	48/52/46/51/50	49.4
**End**	*	AGG	72/74/80/65/48	21/17/24/16/16	19
		AGA	89/75/83/72/50	26/17/25/18/16	20.4
		TAG	58/123/59/108/91	17/29/17/27/29	23.8
		TAA	118/159/116/160/120	35/37/34/40/39	36.8

### 3.6 Phylogenetic analysis

**Fig. 4:**
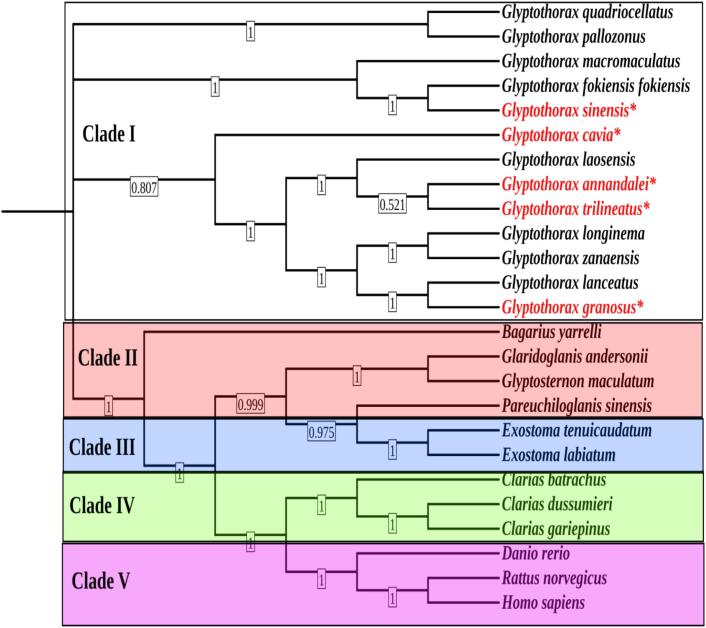
Presents a phylogenetic tree constructed from the complete mitochondrial genome using the Neighbor-Joining (NJ) method with the Kimura 2-parameter (K2P) model and supported by 1000 bootstrap replicates to ensure the reliability of the branching patterns. In this analysis, *Danio rerio* (zebrafish), *Rattus norvegicus* (Norway rat), and *Homo sapiens* (human) were included as outgroups to provide a reference point for evolutionary divergence within the tree.

The phylogenetic analysis based on mitochondrial protein-coding genes revealed that all five target Glyptothorax species clustered within a strongly supported monophyletic group (Clade I; support value = 0.807), along with eight other species of Glyptothorax. Within this clade, *G. granosus, G. annandalei, G. sinensis, G. cavia, and G. trilineatus* grouped closely, suggesting a common evolutionary origin. The subgroup comprising *G. laosensis, G. annandalei, and G. trilineatus* received moderate support (support value = 0.521), indicating their relatively recent divergence. Clade II, comprising *Bagarius yarrelli, Glaridoglanis andersonii, Glyptosternon maculatum,* and *Pareuchiloglanis sinensis*, formed a distinct cluster, separate from the Glyptothorax lineage. Notably, *Glyptosternon maculatum* and *Pareuchiloglanis sinensis* were closely related with a strong support value of 0.999. Clade III consisted of species from the genus *Exostoma (E. tenuicaudatum and E. labiatum)* and was well-supported (support value = 0.975), suggesting the genus forms a distinct and closely related lineage. Clade IV included species from the genus *Clarias (C. batrachus, C. dussumieri, and C. gariepinus)*, which formed a separate group from the sisorid catfishes. Clade V included outgroup species *D. rerio*, *Rattus norvegicus,* and *Homo sapi*ens used to root the tree and establish evolutionary direction. The five Glyptothorax species examined in this study shared close phylogenetic relationships with each other and other Glyptothorax species, while remaining evolutionarily distinct from other sisorid and non-sisorid catfishes. (Fig. 4).

### 3.7 Analysis of Heterogeneous sequence divergence

**Fig. 5:**
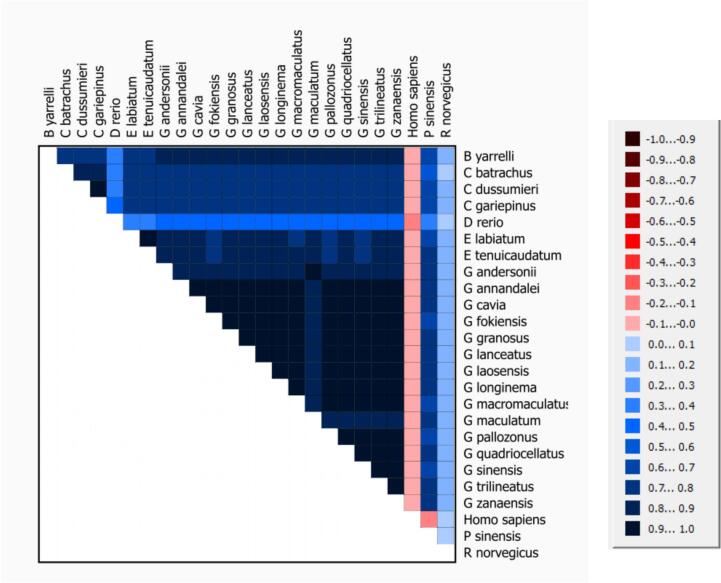
AliGROOVE Analysis of Complete Mitochondrial Genomes for 25 Species:This figure presents the results of an AliGROOVE analysis comparing the complete mitochondrial genome compositions of 25 species. The analysis generates site scores that range from −1 to + 1, where a score of −1 (indicated by red coloring) denotes a significant difference in sequence composition between the genomes, while a score of + 1 (represented by blue coloring) signifies a high degree of similarity in nucleotide composition across the genomes. These scores reflect the relative differences and similarities in the sequence composition of the mitochondrial genomes, providing a visual overview of conserved and divergent genomic regions among the species under study. (For interpretation of the references to colour in this figure legend, the reader is referred to the web version of this article.)

This heatmap, generated by AliGROOVE, visualizes the genetic distance between mitochondrial genomes of various species. Each cell's color intensity represents the genetic similarity or dissimilarity between species pairs: darker blue indicates greater genetic similarity, while lighter shades (up to red or pink) reflect higher genetic divergence. In this heatmap, closely related species, such as those within the Glyptothorax genus (e.g., *G. cavia, G. sinensis, and G. granosus*), show darker blue, indicating high similarity in their mitochondrial genome sequences. On the other hand, species with significant genetic divergence from the Glyptothorax group, such as *Homo sapiens, Danio rerio, and Rattus norvegicus*, display lighter colors in their comparisons with Glyptothorax species, highlighting their distant evolutionary relationship. This visualization helps in identifying genetic closeness and divergence among various freshwater species, showing evolutionary groupings within this dataset (Fig. 5).

### 3.8 Ka/Ks analysis

**Fig. 6:**
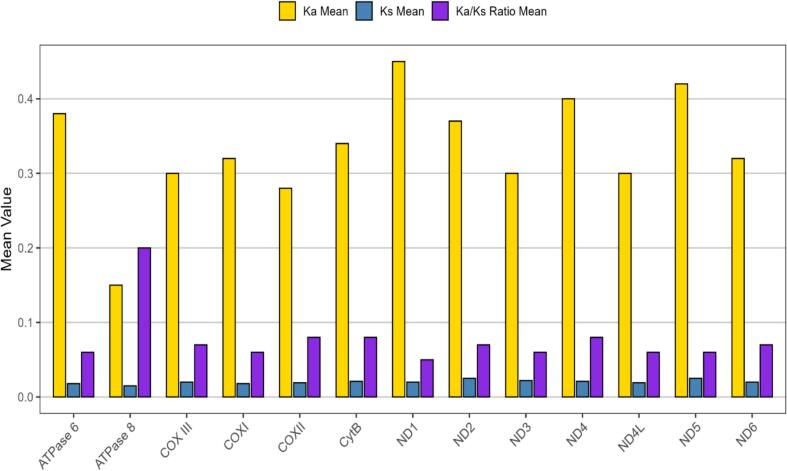
Comparative analysis of Ka (nonsynonymous substitution rate), Ks (synonymous substitution rate), and Ka/Ks ratios for each protein-coding gene (PCG) across the mitochondrial genomes of five Glyptothorax species. This figure highlights the evolutionary dynamics and selective pressures acting on individual genes, with Ka/Ks ratios providing insights into the balance between purifying selection (Ka/Ks < 1), neutral evolution (Ka/Ks = 1), and positive selection (Ka/Ks > 1) across these species.

Synonymous (Ks) and non-synonymous (Ka) substitution rates were calculated for the 13 mitochondrial protein-coding genes (PCGs) across five *Glyptothorax* species to understand their evolutionary constraints. All PCGs exhibited Ka/Ks ratios less than 1, indicating that they are under purifying (negative) selection. Among the genes, ATPase 8 showed the highest Ka/Ks ratio (0.1966), suggesting it is under relatively relaxed selective pressure. This was followed by ND2 (0.0583), ND3 (0.0576), ND5 (0.0490), ND4 (0.0480), and ND6 (0.0447). Moderate purifying selection was observed in ATPase 6 (0.0392), CytB (0.0292), ND1 (0.0238), COXII (0.0232), and COXI (0.0217). The lowest Ka/Ks ratios were recorded in COXIII (0.0152) and ND4L (0.0136), indicating these genes are evolving under strong purifying selection. Overall, the findings reflect evolutionary conservation of mitochondrial genes in *Glyptothorax*, with varying degrees of selective pressure across different genes (Fig. 6).

## 4 Discussion

In phylogenetic investigations, mitochondrial DNA (mtDNA) is an often-used molecular tool due to its straightforward structure, high rate of mutation, maternal inheritance, and restricted recombination. These traits that speed up its growth make mtDNA especially useful for studying genetic diversity and evolutionary connections within and across species.^20^, ^35^, ^36^, ^37^ This study focuses on a comparative mitochondrial analysis of five Glyptothorax species; *Glyptothorax cavia, G. trilineatus, G. annandalei, G. sinensis,* and *G. granosus.* The mitochondrial genome structure of all Glyptothorax species studied was similar to that of other Glyptothorax species and other vertebrates including fish, mice, and humans.^38^ All species containes 13 protein-coding genes (PCGs), two rRNA genes, 22 tRNA genes, and one control region. However, variations in genome length were observed among species, with *G. cavia* at 16,529 bp, *G. trilineatus* at 16,539 bp, *G. annandalei* at 16,541 bp, *G. sinensis* at 16,531 bp, and *G. granosus* at 16,540 bp. Similar size variations were noted in other Glyptothorax species as well.^39^, ^40^, ^41^, ^42^. Size variation in the mt genome may result from insertion or deletion events, duplication of genetic elements, variation in gene spacing, and species-specific adaptations.^43^, ^44^, ^45^. In all five Glyptothorax species studied, the start codon for mitochondrial protein-coding genes is ATG, except for COXI, which uses CTG as its start codon.^42^, ^46^. Mitochondrial genomes often show relaxed codon usage compared to nuclear DNA, permitting alternative start codons like CTG without compromising function..^40^, ^47^ The fact that GTG is consistently used as the COXI start codon across a wide variety of fish species raises the possibility that this variation reflects the gene's ancient origin and evolutionary conservation..^18^ .

Five Glyptothorax species showed similar sequence patterns with other fishes, though some slight differences were also detected. For instance, the stop codons TAA, Txx and Tax only been observed in all Glyptothorax species. Where some vertebrate fish species been reported only Txx..^48^, ^49^ One reason for stop codon variation in mitochondrial genomes is genome streamlining, which can potentially boost transcriptional or translational efficiency. Studies suggest that certain fish species use alternative stop codons, such as TAG or TGA, instead of the typical TAA.^50^, ^51^ This variation may be shaped by factors like mutational biases and the availability of specific tRNAs, both regulated by the genome.^52^ These adaptations can optimize protein synthesis to meet the high-energy demands of fish, which is particularly critical for efficient mitochondrial function and rapid energy production.^53^ Distinct stop codon patterns in mitochondrial genomes can aid in differentiating closely related species, often representing evolutionary differences unique to specific lineages. Along with other mitochondrial DNA markers, these variations act as reliable indicators for species identification and lineage tracing.^2^, ^54^. The fact that these vital mitochondrial genes are conserved across species emphasizes both their evolutionary significance and critical functional relevance..^55^, ^56^ The use of these stop codons TAA, TAG, and single T appears to be a distinctive feature in Glyptothorax mitochondrial genomes, as all five studied species exhibit this pattern. This flexibility in stop codon usage is a well-known characteristic of mitochondrial genomes, reflecting their relaxed codon constraints and unique translational machinery. Such findings help clarify the evolutionary and functional dynamics of mitochondrial gene expression in Glyptothorax and related fishes.^20^, ^49^,^57^.

Particularly with regard to the percentages of AT and GC content, the mitochondrial genome data of the five Glyptothorax species varied significantly at the nucleotide composition level. Adenine (A) and thymine (T) nucleotides were preferred in the mitochondrial genome of the *G.sinensis,* which had the highest AT concentration of the five species. There are a number of reasons why some species' mitochondrial genomes have higher AT content. Particularly in genomes that mutate quickly, mutational biases frequently favour A and T nucleotides, increasing the AT ratio. Additionally, under particular environmental conditions, such lower temperatures, where AT bonds need less energy to separate, AT-rich sequences may provide adaptive advantages and contribute to genome stability..^58^, ^59^ AT-rich genomes may also be preferred by selection pressures on mitochondrial function to improve transcription and translation efficiency, especially in species with high energy requirements. Additionally, a higher AT content in specific species is reinforced by the accumulation of AT-biased mistakes over time due to mitochondria's poor DNA repair mechanisms. Conversely, the *G. annandalei* reported the highest GC content than other Glyptothorax species. Variations in GC content across mitochondrial or nuclear genomes can be characteristic of specific taxonomic groups, making it a useful marker in distinguishing between closely related fish species..^22^, ^60^ The mitochondrial genome of G*lyptothorax* species contains two types of ribosomal RNAs, 12S rRNA and 16S rRNA, which are flanked at the 5′ end by the tRNA Phe gene and the rRNA Val gene, and at the 3′ end by the tRNA Val and tRNA Leu genes. This arrangement is consistent across other *Glyptothorax* spp and has also been observed in various other fish species..^39^, ^41^, ^42^, ^46^, ^61^ The rRNA and mRNA genes are separated from tRNA genes in mitochondria. Processing these tRNA genes aids in the accurate maturation of rRNAs and mRNAs, both of which are necessary for the production of proteins in the mitochondria. The correct processing and functionality of the rRNAs for their function in the mitochondrial ribosome are guaranteed by this mechanism^62^, ^63^. All other tRNAs, except for trnS1 (Ser), which did not have the D arm, were shown to have the characteristic cloverleaf secondary structure. This secondary structure is usually essential for the stability and functionality of tRNA^64^.Amino acid recognition and protein synthesis may be greatly impacted by any changes or removals of crucial secondary structure regions.^65^, ^66^ Accurate prediction and recognition of these secondary structures from amino acid sequences have improved over time, with modern computational methods now able to identify structural features and their roles in protein function with increasing reliability.^66^ These advances highlight the strong correlation between sequence, secondary structure, and protein function, emphasizing that even subtle changes in secondary structure can have profound effects on protein synthesis and biological activity.

In DNA sequences, AT-skew and GC-skew are often used as indicators of strand asymmetry and nucleotide composition patterns.^67^ The mean AT-skew and GC-skew values across protein-coding genes (PCGs) in Glyptothorax species reveal distinct patterns in nucleotide composition. Generally, most genes show an AT-rich composition, with ATPase 8 and ND2 displaying a strong preference for adenine, while ND6 has a marked preference for thymine. Similarly, GC-skew values suggest a cytosine bias across most genes, except for ND6, which exhibits a strong guanine preference, likely due to its transcription from the opposite strand compared to other mitochondrial genes. These skews indicate functional and structural demands within the mitochondrial genome, with evolutionary adaptations potentially favouring these nucleotide asymmetries to optimize gene function across these species. Genes with strong nucleotide biases, like the guanine-rich ND6 and adenine-favoring ATPase 8, may reflect selective pressures that optimize gene stability, expression, or replication efficiency in mitochondrial functions.^68^ AAA for lysine, CAA for glutamine, and TGA for tryptophan were the most commonly utilized codons in all five Glyptothorax species. The amino acids with the most codons were lysine (AAA), leucine (CTA), and others. The highest leucine codon counts were found *in G. granosus, G. annandalei, and G. sinensis*, with the exception *of G. trilineatus*. The codons with the lowest frequency across all Glyptothorax species were TCA for serine, GCG for alanine, and ACG for threonine. Several species' mitochondrial genomes showed significant codon usage biases, which subjected the gene to different selection pressures and allowed for the prediction of gene function.^69^, ^70^, ^71^.

Interesting new information about the *Glyptothorax* genus is revealed by the phylogenetic study. Due to genetic adaptations that have maintained similarities in their nuclear and mitochondrial DNA over time, as well as shared environmental pressures and geographic proximity*, G. cavia and G. trilineatus* have formed a distinct lineage, indicating a close evolutionary relationship. These two species could share a recent common ancestor's genetic features because they inhabit similar ecological niches or are subject to similar selection pressures.^72^, ^73^ On the other hand, the higher levels of genetic diversity shown in *G. annandalei*, *G. sinensis,* and *G. granosus* most likely stem from alternative evolutionary paths influenced by environmental differences, geographic isolation, or special adaptive traits. This divergence implies that these species split off from the common ancestor earlier and have accumulated as a result of prolonged separate development.^42^, ^46^ The distinct lineages of *P. sinensis*, *G. maculatum*, *G.andersonii*, and *B. yarrelli,* separate from the Glyptothorax group, are likely due to adaptations to unique environmental conditions and specialized ecological niches. Differences in habitat factors like water flow, altitude, temperature, and food availability have driven specific evolutionary pressures. Over time, these species developed unique physiological and behavioral traits, optimizing survival in their respective environments. This has led to genetic divergence and the formation of separate evolutionary paths^74^ The distinct clades formed by *Glyptothorax* species, separate from higher vertebrates like humans and rats, highlight their long evolutionary divergence due to fundamental differences in lineage that arose early in the vertebrate evolutionary timeline. As these lineages adapted to vastly different environments and biological roles, *Glyptothorax* species retained characteristics aligned with aquatic life, particularly specialized adaptations within freshwater habitats. When compared to other fish, such as zebrafish and *Clarias* catfish, *Glyptothorax* species demonstrate closer evolutionary relationships to each other, likely due to their shared genetic, ecological, and morphological traits specific to certain freshwater environments. Meanwhile, the distinct clades formed by species like zebrafish, *Clarias*, *P. sinensis*, and *G. maculatum* reflect their evolutionary adaptations to different ecological niches or habitats, driving their divergence from the *Glyptothorax* lineage over time.

Purifying selection was evident in all five species of Glyptothorax, according to synonymous and non-synonymous substitution studies for 13 protein-coding genes (PCGs) (Ka/Ks < 1). which means that nonsynonymous substitutions (amino acid-changing mutations) are being selected against, while synonymous substitutions (silent mutations that do not alter amino acids) accumulate more frequently. This is because nonsynonymous changes can disrupt protein structure or function, potentially reducing an organism's fitness^75^, ^76^, ^77^.

## 5 Conclusion

This study presents a comprehensive comparative mitogenomic analysis of five Glyptothorax species *G. cavia, G. trilineatus, G. annandalei, G. sinensis, and G. granosus*. All species shared the conserved vertebrate mitochondrial genome structure, comprising 13 protein-coding genes (PCGs), 22 transfer RNAs (tRNAs), two ribosomal RNAs (rRNAs), and a control region. Despite this conserved architecture, notable interspecific variations were observed in genome length, nucleotide composition, gene arrangement, codon usage, and tRNA secondary structures. Nucleotide composition analyses revealed species-specific AT and GC content, with *G. sinensis* exhibiting the highest AT bias, and *G. annandalei* displaying the highest GC content. Strand asymmetry analyses showed consistent positive AT-skews and negative GC-skews across most genes, with exceptions such as ND6, which showed a distinct guanine bias. Codon usage was skewed, with a strong preference for AAA (Lys), CAA (Gln), and TGA (Trp), and a notably low usage of TCA (Ser), GCG (Ala), and ACG (Thr), indicating selection-driven codon bias. tRNA secondary structure analysis confirmed the typical cloverleaf configuration across most genes, except for trnS1 (Ser), which lacked the dihydrouridine (D) arm. Phylogenetic analysis based on mitochondrial PCGs revealed a well-supported monophyletic grouping of the target Glyptothorax species, highlighting their close evolutionary relationships and supporting species-level resolution. Sequence divergence heatmaps and Ka/Ks analysis further corroborated the phylogenetic clustering and evolutionary constraints, with all PCGs exhibiting Ka/Ks ratios < 1, indicative of strong purifying selection acting to preserve mitochondrial gene function. These findings contribute valuable insights into mitochondrial genome evolution and species differentiation within *Glyptothorax*. However, given the genus's ecological diversity and wide geographic distribution, there is a clear need to expand mitogenomic studies to additional *Glyptothorax* species. Such efforts are essential to refine phylogenetic relationships, uncover cryptic diversity, and better understand the evolutionary dynamics within this diverse group of sisorid catfishes.

## CRediT authorship contribution statement

**Somasundaram Iyyappan:** Writing – original draft, Resources, Methodology, Formal analysis, Data curation. **Suvadip Ghara:** Formal analysis, Writing – review & editing. **Irfan Ahmad Bhat:** Writing – review & editing, Validation, Supervision, Resources. **Irfan Ahmad Khan:** Resources, Supervision, Validation, Writing – review & editing. **Mohd Ashraf Rather:** Writing – review & editing, Validation, Supervision, Project administration, Methodology, Conceptualization.

## Funding

This study was not funded by any agency**.**
